# Cumulative residential greenness and childhood body mass index

**DOI:** 10.1097/EE9.0000000000000421

**Published:** 2025-09-25

**Authors:** Jo Davies, Lucy J. Griffiths, Theodora Pouliou, Rowena Bailey, Richard Fry, Ronan A Lyons, Gareth Stratton, Amy Mizen

**Affiliations:** a Population Data Science, Swansea University Medical School, Swansea, United Kingdom; b School of Public Health and Preventive Medicine, Monash University, Melbourne, Victoria, Australia; c Research Centre in Applied Sports, Technology, Exercise and Medicine, Swansea University, Swansea, United Kingdom

## Abstract

**Background:**

Childhood obesity is a complex and multifaceted public health issue. Several studies have found that children living in greener neighborhoods have a lower body mass index (BMI); however, evidence on longitudinal exposure remains limited. This study examined the relationship between Enhanced Vegetation Index (EVI), green space, and children’s weight status using linked environmental and national health data.

**Methods:**

We derived annual EVI values from Landsat 8 satellite imagery (30 m resolution) within 300 m of a child’s residence in Wales from 2008 to 2019. Mean EVI exposure was calculated for the 4 years preceding BMI measurement. We utilized 2017 Ordnance Survey Open Greenspace data to identify green spaces within 800 m of a child’s residence. BMI obtained from the Child Measurement Programme for Wales (2012/13 to 2018/19) for children aged 4–5 years was used to define healthy versus overweight/obesity. We used logistic regression to evaluate associations between residential greenness, green spaces, and childhood weight status.

**Results:**

The final cohort consisted of 200,237 children. A one-unit increase in EVI was associated with a 20% higher likelihood of being overweight or obese (OR = 1.20, 95% CI = 1.05, 1.37). For every additional green space within 800 m, the likelihood of having an unhealthy weight increased by 0.3%.

**Conclusions:**

Our findings suggest that EVI and access to green spaces should be interpreted with care, as they may not capture how young children interact with nearby green environments. Future work investigating the impact of greenness and greenspace on child weight status should use measures tailored to more accurately represent age-specific behaviors.

**What this study adds**

This is the first study to use novel data linkages of geospatially derived measures of the built and natural environment to explore associations between objective measures of residential greenness (Enhanced Vegetation Index), green space, and childhood weight status in a nationally representative cohort of 200,237 children aged 4–5 years old. Findings from this study highlight the complex interplay between environmental and socioeconomic factors in shaping children’s weight status and suggest that while the Enhanced Vegetation Index captures general vegetation cover, it may not adequately reflect how young children interact with green spaces in their immediate residential environment.

## Introduction

Childhood obesity is a complex and multifaceted public health issue, carrying substantial short- and long-term consequences for individual health and well-being.^1^ The prevalence of obesity among children has increased substantially, affecting an estimated 159 million individuals aged 5–19 worldwide.^2^ In the UK alone, approximately 24% of children aged 4–5 years old are living with overweight or obesity, with a greater prevalence in more deprived areas compared to the least deprived areas.^3^

The etiology of childhood obesity is influenced by a multitude of factors, including genetic predisposition, dietary habits, physical activity levels, sedentary behavior, and psychosocial and environmental exposures.^4^ Among these factors, the built environment and surrounding ecological characteristics have emerged as important determinants shaping obesity risk. Obesogenic environments—“the collective physical, economic, policy, and sociocultural surroundings, opportunities, and conditions that promote obesity”—are key drivers.^5^ Identifying and targeting modifiable aspects of these environments can benefit entire communities.

In recent years, attention has been drawn to modifiable aspects of the built environment, including residential greenness and its association with noncommunicable diseases such as obesity.^6–8^ However, there is much heterogeneity in how studies define objective measures of residential greenness. For example, vegetation indices based on remotely sensed data from satellite imagery^9–11^ have been widely used as measures of residential greenness.^12^ Defining residential greenness using vegetation indices such as the Enhanced Vegetation Index (EVI) allows for longitudinal measurements across large spatial regions to be calculated. Greater vegetation index values are generally interpreted as representing greater access to green space by area because vegetation indices are indicators of green vegetation, which is inherently what makes a green space. However, EVI is a dimensionless measure of green reflectance, and it has been demonstrated that EVI may not be linearly associated with 2-dimensional measures of access to green space nor capture green space signatures in three dimensions.^13^ Thus, higher EVI scores do not necessarily equate to increased accessibility to publicly available green areas.^14^ Defining residential greenness using vector-based data allows for accurate boundaries of formal green spaces to be measured. However, these datasets are generally captured by mapping agencies and local governments and are subject to licensing agreements. These data also tend to be cross-sectional. Recent studies acknowledge the difference between these objective measures^10,15,16^ but there have been few attempts to understand whether different definitions of residential greenness yield different associations with childhood body mass index (BMI).

Several studies have found that children living in greener neighborhoods have a healthier weight status;^6–8^ however, it remains unclear whether this has a longitudinal impact on the weight status of children as they grow up. The evidence is inconsistent, with varying definitions of residential greenness contributing to the complexity.^15^ Few studies focus on younger children^17,18^ and so understanding how young children use green space and its impact on childhood weight status is still in its infancy. In addition, the mechanisms underlying associations of green space with overweight and obesity remain poorly understood, although they may encompass factors such as increased opportunities for physical activity, stress reduction, and increased social connectedness, lower crime, as well as less antisocial behavior.^19^

This study examined the association between two objective measures of residential greenness and children’s weight status, adjusting for gender, deprivation, and urbanicity. We used two widely used objective measures of residential greenness: average EVI 300 m around household location and count of greenspaces within 800 m of household location. This is the first study to explore associations between objective measures of residential greenness and a national population of children aged 4 and 5 years old from Wales, United Kingdom, using national surveillance BMI data. We hypothesize that children with a greater exposure to residential greenness will be more likely to have a healthy weight.

## Material and methods

### Study design

This population-level, retrospective cross-sectional observational study utilized routinely collected data held in the Secure Anonymized Information Linkage (SAIL) Databank, hosted at Swansea University.^20,21^ Swansea University is a leading research institution in Wales, with extensive expertise in health informatics and data linkage. The SAIL Databank provides a secure, privacy-protecting environment for accessing and analyzing anonymized, individual-level health, socioeconomic, and environmental data from multiple sources across Wales, enabling robust population-level research. For each data source within the SAIL Databank, personal identifiable data has been removed and replaced with an anonymized linkage field (ALF) to enable linkage of records from various sources. SAIL also uses a residential anonymized linkage field (RALF) to link individuals with their place of residence. The anonymization and linkage methodology are described elsewhere.^21–24^

### Data sources and population

The child measurement programme (CMP) for Wales provides robust public health surveillance data on the BMI of school-aged children and is used to estimate levels of overweight and obesity at local and national levels. Since 2011, heights and weights of children in reception class of state-maintained schools have been collected via the CMP. Measurements are carried out using a standardized protocol by trained school health team members across Wales.

**Figure 1.**
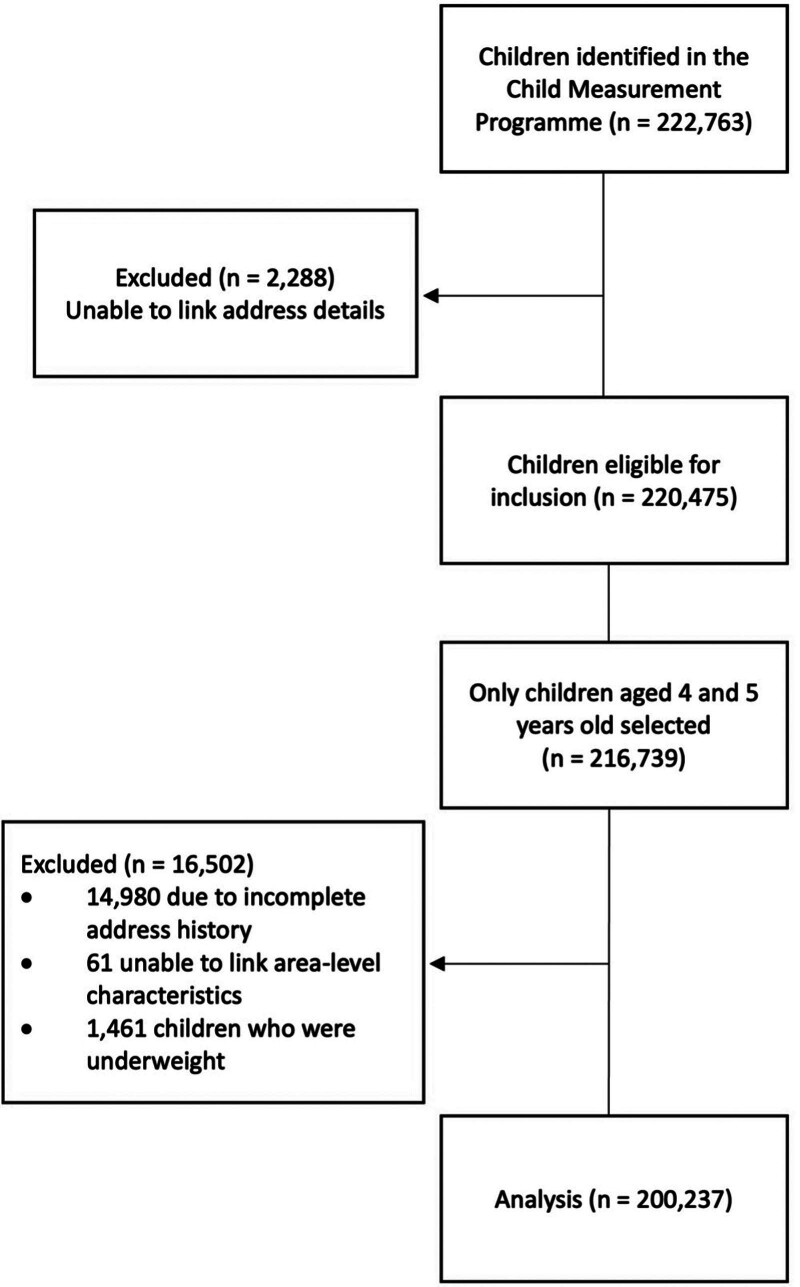
Cohort consort diagram.

The CMP contains information on 222,763 children between 2012/13 and 2018/19; however, 17,329 (8%) children were excluded due to missing address details (Figure 1). We excluded 2,288 children as we were unable to link their address details, 3,736 children who were not within the age inclusion criteria of 4–5 years of age, 14,980 children due to incomplete address history, and 61 children as we were unable to link EVI and green space data. We also excluded 1,461 children who were underweight due to small numbers. The final cohort consisted of 200,237 children aged 4–5 years old.

The Welsh Demographic Service Dataset (WDSD) contains address records and demographic information for people accessing NHS services in Wales and registered to a Welsh address. WDSD was used to derive age, gender, socioeconomic deprivation quintile, and residential information for the children within a CMP record. An annual census date of 1st June was used to determine at which residence each child was registered in the 4 years before their measurement date. This date was chosen to coincide with periods of peak greenness obtained from satellite data. Any children who did not have a Welsh residence recorded for each of the 4 years preceding their BMI measurement were recorded as having an incomplete address history and were subsequently excluded from the analysis.

EVI work reported in this paper was conducted as part of a wider longitudinal study called the Green-Blue Spaces project,^14,16,25,26^ where a national-level annual exposure variable for 1.49 million households over 11 years (2008–2019) using satellite-derived EVI was developed. We utilized Landsat 8 satellite imagery with a 30-meter resolution to derive the EVI values for all residential addresses in Wales. The EVI values were calculated using the red, blue, and near-infrared (NIR) reflectance bands from the Landsat imagery, processed via the vegetation index Geographic Resources Analysis Support System (GRASS) tool in Quantum Geographic Information system (QGIS).^27^ A 300 m Euclidean buffer around each residential dwelling was used to coincide with World Health Organization guidelines for green space access^28^ (full details of the EVI estimation methodology are described elsewhere).^14,16^ EVI data were calculated annually from 2008 to 2019.

We obtained green space data from the 2017 Ordnance Survey Open Green Spaces Dataset^29^ to capture the total number of formal green spaces within 800 m of a child’s home.

### Outcome

Heights and weights recorded in the CMP data were used to calculate the BMI of each child. BMI status was then categorized according to the UK1990 clinical reference standards,^30^ into four mutually exclusive groups: “underweight” (BMI <second centile), “healthy weight” (≥second to <91st centile), “overweight” (≥91st to <98th centile), or “very overweight” (≥98th centile) (sometimes referred to as obese) based on alignment with sex- and age-standardized z-scores. Z-scores more than five standard deviations away from the mean were discarded. The BMI z-score categories were then used to derive a binary measure indicating weight status, with 0 being a healthy weight and 1 being an unhealthy weight (i.e., overweight or obese) to align with public health standards. This categorization facilitates interpretation in terms of overweight risk and reflects the clinical relevance of weight categories in early childhood. Children who were classed as underweight were excluded from the study due to small numbers.

### Residential greenness exposure variables

#### Enhanced Vegetation Index

To assign a neighborhood EVI exposure value to each residential address location in Wales (n = 1.49 million), we created a Euclidean buffer of 300 m around each child’s residence in Wales. Annual EVI values were derived for each child’s residential address using satellite imagery for the 4 years preceding their BMI measurement year. These four values were then averaged to create a single exposure variable representing longer-term greenness exposure. For children not registered at an address on 1st June, the EVI value was derived from the first recorded address thereafter. The EVI values were linked to the residential address closest to the time of BMI measurement and were assumed to be stable across the 4 years. EVI values range from −1 (water) to +1 (vegetation), with healthy vegetation values typically found in the 0.2–0.8 range.^31^ Average EVI was modeled as a continuous variable in the statistical analyses, where each unit increase represents a one-unit change in EVI (i.e., 0.2–0.3).

### Number of green spaces

Green spaces classified in the Open Green spaces Dataset as “play space,” “public park or garden,” or “playing field” were extracted for our study. These classifications were used as they were deemed to be most appropriate for the age of our cohort. Green spaces with a pedestrian or vehicle access point within 800 m of a child’s residence were included, as this proximity has been defined as walkable for children of this age^32^ and is widely applied in the literature. Green spaces with multiple access points were only counted once. The total count of green spaces within 800 m of a child’s residence was calculated annually for the 4 years before BMI measurements being taken to determine the average number of green spaces each child had access to during that period. Counts were used to determine the cumulative opportunity for children and their parents to engage in physical activity in green space within their local neighborhood (800 m or approximately 15 min walk). Number of green spaces was modeled as a continuous variable in the final analyses, where each unit increase represents one additional green space.

### Covariates

Age (in years) was calculated at the date of the BMI measurement using the week of birth from the WDSD. Gender was also obtained from the WDSD as a dichotomous variable representing males and females. The lower layer super output area (LSOA) of residence at the time of BMI measurement was used to derive LSOA level characteristics (i.e., urban/rural). Deprivation quintile was obtained from the 2019 Welsh Index of Multiple Deprivation (WIMD) at the LSOA level, with 1 being the most deprived quintile and 5 being the least deprived. WIMD 2019 was also used to derive urban/rural characteristics.

### Statistical analyses

Our descriptive analysis included summary measures of our outcome variable—weight status—as well as an average of the exposure variables within Wales. Further, socioeconomic characteristics of the participants were also described. Categorical variables were presented as numbers and percentages within each group, while continuous variables were summarized using means and standard deviations.

We used logistic regression to evaluate whether exposure to residential greenness was associated with weight status. We exponentiated the model coefficients and reported the adjusted odds ratio (OR) along with 95% confidence intervals (95% CI). We interpreted statistical significance using a *P* value < 0.05.

## Results

### Descriptive analysis

The sample consisted of 101,966 (51%) boys and 98,271 (49%) girls. Forty-two percent were 4 years old, and approximately 58% were 5 years old. At the time of BMI measurement, children in the cohort predominantly lived in urban areas (72%), and over a quarter lived in areas with the highest levels of deprivation. Just over 80% of the cohort were recorded as a healthy weight in the CMP, while 18% were recorded as overweight or obese (Table 1). Just over 20% of children living in the most deprived quintile were of an unhealthy weight compared with only 14% in the least deprived quintile.

**Table 1.** Descriptive statistics of the sample (n = 200,237), Wales, UK, 2012/13–2018/19

Characteristic	Healthy weight (%)	Unhealthy weight^a^ (%)
N	163,640 (81.7)	36,597 (18.3)
Sex		
Male	82,920 (81.3)	19,046 (18.7)
Female	80,720 (82.1)	17,551 (17.9)
Age		
4	69,058 (81.3)	15,853(18.7)
5	94,582 (82.0)	20,744 (18.0)
Urbanicity		
Rural	45,296 (81.6)	10,243 (18.4)
Urban	118,344 (81.8)	26,354 (18.2)
Deprivation quintile		
1 Most deprived	40,486 (79.3)	10,553 (20.7)
2	34,170 (80.4)	8,317 (19.6)
3	29,704 (81.5)	6,732 (18.5)
4	28,966 (82.7)	6,047 (17.3)
5 Least deprived	30,314 (86.0)	4,948 (14.0)
	Mean (SD)	Mean (SD)
EVI	0.3 (0.1)	0.3 (0.1)
Number of greenspaces	14.5 (13.1)	15 (13.3)

a Overweight or obese.

**Figure 2.**
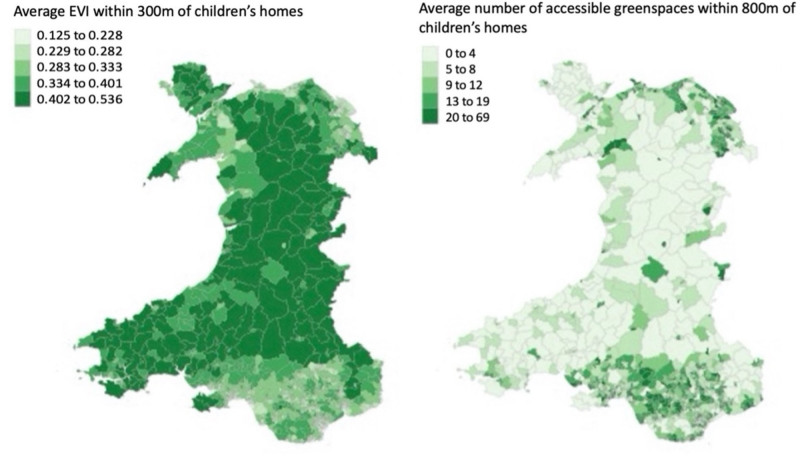
Average EVI and average number of green spaces surrounding children’s homes by Lower layer Super Output Area, Wales, UK, 2012/13–2018/19.

In terms of environmental characteristics, the highest EVI values were recorded in rural parts of mid and West Wales (Figure 2) with EVI values decreasing towards more urban areas. The lowest mean EVI value recorded within 300 m of children’s homes was 0.03, while the highest was 0.75. The average EVI value for the cohort was 0.3.

Average EVI and average number of green spaces surrounding children’s homes by LSOA are shown in Figure 2. Seventeen percent of children had no access to age-appropriate green space within 800 m of their homes (S-Table 1; https://links.lww.com/EE/A373).

### Enhanced Vegetation Index—adjusted analysis

When adjusting for covariates, we found that for every unit increase in EVI, children were 20% more likely to be overweight or obese (Table 2). Children in the least deprived quintile were 38% less likely to have an unhealthy weight compared with those living in the most deprived areas. Also, children were less likely to be overweight or obese if they were 5 years of age (OR = 0.96; 95% CI = 0.94, 0.98) compared to 4-year-olds; or were living in urban areas (OR = 0.96; 95% CI = 0.93, 0.99) compared to those living in rural areas. All associations were significant at the 0.05 level of significance.

**Table 2.** Differences in children’s weight status (healthy weight vs. overweight or obese) based on average EVI, Wales, UK, 2012/13–2018/19

	Unadjusted		Adjusted	
	Odds ratio^a^ (95% CI)		Odds ratio^a^ (95% CI)	
Average EVI	1.03 (0.92 - 1.16)		1.20 (1.05 - 1.37)	
Age (years)				
4	-		-	
5	0.96 (0.93, 0.98)		0.96 (0.94, 0.98)	
Sex				
Male	-		-	
Female	0.95 (0.93, 0.97)		0.95 (0.92, 0.97)	
Deprivation quintile (WIMD)				
1 Most deprived	-		-	
2	0.93 (0.90, 0.96)		0.93 (0.90, 0.96)	
3	0.87 (0.84, 0.90)		0.85 (0.82, 0.88)	
4	0.80 (0.77, 0.83)		0.78 (0.75, 0.81)	
5 Least deprived	0.63 (0.60, 0.65)		0.62 (0.60, 0.64)	
Urban/rural				
Rural	-		-	
Urban	0.98 (0.96, 1.01)		0.96 (0.93, 0.99)	

a Odds ratio per 1 unit increase in EVI (i.e., 0.2–0.3). Outcome is overweight or obese; reference category is healthy weight.

We also explored the association between average EVI for each weight category separately (S-Table 2; https://links.lww.com/EE/A373) and found that healthy weight had the strongest positive association with EVI, indicating that for every unit increase in EVI, children were 20% more likely to have a healthy weight. Overweight and obese categories showed negative associations with EVI. However, these associations were not significant.

For EVI exposure, we also conducted stratified analyses by urban/rural categorization (S-Table 3; https://links.lww.com/EE/A373) and by gender (S-Table 4; https://links.lww.com/EE/A373). Findings demonstrated that in urban areas, a higher average EVI significantly increases the odds of being overweight or obese, while the effect is not significant in rural areas. In particular, in urban areas, children are 47% more likely to be overweight or obese for every unit increase in average EVI. When stratified by gender, results showed a statistically significant association for males but not for females. However, a formal test for interaction indicated no evidence that the association differed by gender (*P* > 0.05).

### Number of green spaces—adjusted analysis

For every additional green space within 800 m of a child’s home, the likelihood of having an unhealthy weight increased by 0.3% (Table 3). Children living in the least deprived areas had lower odds of being overweight or obese compared with those living in the most deprived areas. In addition, girls and those living in urban areas were less likely to be overweight or obese compared with boys and those living in rural areas.

**Table 3.** Differences in children’s weight status (healthy weight vs. overweight or obese) based on average number of green spaces, Wales, UK, 2012/13–2018/19

	Unadjusted		Adjusted	
	Odds ratio^a^ (95% CI)		Odds ratio^a^ (95% CI)	
Average number of green spaces	1.00 (1.00, 1.00)		1.00 (1.00, 1.01)	
Age (years)				
4	-		-	
5	0.96 (0.93, 0.98)		0.96 (0.94, 0.98)	
Sex				
Male	-		-	
Female	0.95 (0.93, 0.97)		0.95 (0.92, 0.97)	
Deprivation quintile (WIMD)				
1 Most deprived	-		-	
2	0.93 (0.90, 0.96)		0.93 (0.90, 0.96)	
3	0.87 (0.84, 0.90)		0.86 (0.83, 0.89)	
4	0.80 (0.77, 0.83)		0.79 (0.76, 0.82)	
5 Least deprived	0.63 (0.60, 0.65)		0.63 (0.60, 0.65)	
Urban/rural				
Rural	-		-	
Urban	0.98 (0.96, 1.01)		0.94 (0.92, 0.97)	

a Odds ratio per 1 additional green space within an 800 m Euclidean buffer, averaged over the 4 years before BMI measurement. Outcome is overweight or obese; reference category is healthy weight.

## Discussion

### Summary of key findings

This study examined the association between two objective measures of residential greenness and children’s weight status, adjusting for gender, deprivation, and urbanicity. This is the first study to explore associations between objective measures of residential greenness and a national population of young children from national surveillance BMI data. Contrary to our hypothesis, findings demonstrate that children were more likely to be overweight or obese if they lived in areas with higher average EVI. We also found that increasing the number of green spaces increased the likelihood of a child being overweight or obese. All associations were significant when adjusted for gender, deprivation, and urbanicity.

### Comparison with previous studies

In general, while the existing evidence affirms beneficial impacts of green space on physical and mental health, much remains to be learned about the specific pathways and functional form of such relationships, and how these may vary by context, population groups, and health outcomes.^8,19^ This is confirmed by a recent review of 21 studies where mixed results were observed for the association between green space and children’s weight-related behaviors or outcomes.^33^ In particular, while most studies found that green space was negatively associated with weight status, some did report either opposite or null associations. For example, Picavet et al^34^ in a study carried out in the Netherlands reported weak support for the hypothesis that greater residential greenness positively impacts most health indicators (e.g., blood pressure, diabetes, overweight/obesity, mental health disorders etc.). Wilhelmsen et al^35^ explored associations between green area in school neighborhoods and overweight and obesity among 10,527 Norwegian adolescents and found that the percentage of overweight and obese adolescents increased significantly when the percentage of green areas in the participants’ surroundings increased. Further, a small study of 1,489 children living in Lithuania by Petraviciene et al^17^ found that while children living in areas with less residential greenness (NDVI-100 m) had significantly higher ORs of being overweight/obese, this association was not significant for the NVDI-300 m and NVDI-500 m buffer sizes, indicating that NDVI values are potentially sensitive to certain types and amounts of vegetation within various buffer zones and therefore, interpretation of findings and policy-decisions require careful evaluation of greenness measures. Finally, a recent systematic review included 51 studies to explore the association between green space and overweight and obesity,^7^ yet, only two explored UK children’s data.^36,37^ Both of those studies used the Millennium Cohort Study data and self-reported measures of greenness access and exposure. However, the findings were mixed, with Schalkwijk et al^36^ reporting a statistically significant association between low levels of green space and childhood overweight/obesity, while van der Zwaar^,37^ reported no significant association between green space and children’s weight.

### Strengths and limitations

Our study has several strengths, specifically the linkage of a national population dataset with objectively measured environmental data, enabled through the SAIL Databank. To the best of our knowledge, this is the first time that CMP data from Wales have been linked to objectively measured environmental data to examine the potential associations between residential greenness as well as deprivation and children’s weight status. The inclusion of objective measurements for both outcome and exposure adds validity to our analysis, as these measurements reduce recall and reporting biases commonly associated with self-reported data. Focusing on Wales also provides a geographically specific context, which can be particularly valuable for policymakers and stakeholders in the region. Applying an objective measure of residential greenness based on an average exposure for each child over the 4 years preceding their BMI measurement provides a dynamic method for studying the relationship. Such a dynamic green space exposure assessment strategy, which accounts for changes in green space over time, can also be used to capture more accurate green space exposure. Satellite-derived measures such as EVI offer the opportunity to calculate objective and uniformly measured exposures of exposure to green space.

We also acknowledge that this study has several limitations. Objectively measured height and weight, equipment calibration, participant compliance, and the specific measurement protocols used can introduce measurement errors that affect data quality. The EVI buffers were Euclidean distances, and the access buffers were calculated for a 300 m network distance. Although the buffers are not a like-for-like comparison in shape, at this scale, we are confident that this did not significantly impact our results. Further, using the same method of measuring residential greenness as a standard approach will allow for comparison with other studies, as has been highlighted in recent reviews as the way forward in understanding the association between greenness and childhood weight status.

We assumed residential stability across the 4-year exposure window and did not account for potential residential moves. This may have introduced some exposure misclassification for children who moved during this period. Additionally, WIMD and urban/rural classifications were assigned from 2019 data, and we did not examine earlier years’ classifications. Despite this, these measures show limited change over time, especially during the short exposure window used for this study.

We used 2017 OpenStreetMap data for the green space measure, as this was the only available dataset at the point of analysis to allow linking with the CMP (2012–2019) data within the SAIL Databank; however, the difference in years is small, and we did not expect any changes in the environment that would impact the findings of this study. While we modeled the number of green spaces as a continuous variable to retain full exposure variability, we acknowledge that the resulting effect estimates may be difficult to interpret due to the small unit of change. Future research could consider using standardized units or categorizing green space counts to explore potential nonlinear associations and improve interpretability.

Satellite-derived measures such as EVI are currently the only way to calculate internationally comparable measures of the built environment. The challenges of working with satellite data include spatial and temporal resolution, cloud cover, shadows cast by buildings in dense urban areas, and missing smaller urban green spaces such as trees and pocket parks found in urban areas. Furthermore, finding appropriate cloud-free satellite data at the correct time of year can be particularly challenging for northern-hemisphere climates. Satellite data processing includes adjustments and masks to mitigate some issues of cloud cover, but it remains challenging to work with at a national level and may lead to exposure misclassification. Finally, we acknowledge that unmeasured confounding may remain. In particular, we lacked data used in the “classic” energy equation (e.g., diet and exercise), which, along with genetics and household income, might explain variations in both exposure to greenness and children’s weight status.

### Implications of the findings

While several studies have found that children living in greener neighborhoods have a healthier weight status, the evidence is inconsistent, especially regarding the longitudinal impact of residential greenness on the weight status of children. While the association between children’s weight status and residential greenness does not permit us to draw solid conclusions about the health-promoting effects of living in greener environments, it emphasizes the complexity of this relationship and suggests that other features of the environment should also be considered when attempting to explain the variance in children’s weight status and residential greenness exposure.

Measuring residential greenness exposure is a complex task. While EVI provides an objective measure of vegetative density, it does not necessarily capture access to publicly available or usable green space. As Mizen et al^14^ highlighted, higher EVI scores should be interpreted with caution, as they may not reflect the presence of accessible green spaces where children can engage in unstructured physical activity, which would potentially have a positive impact on weight status. Their findings highlight the importance of treating EVI and green space access as conceptually distinct exposures, given their potential to influence child health through different mechanisms.

This distinction is further supported by Klompmaker et al^15^ who found that associations between green space and overweight or physical activity varied considerably depending on how green space was defined—particularly in more urban populations.^15^ Further, according to a study conducted in the UK, applying a cross-sectional survey to a random sample of 2,079 working-age adults, nature-relatedness was the strongest predictor for both visit frequency to local green space and meeting physical activity guidelines.^38^

Further, residential greenness exposure at different points across the life course may have a different impact with greater longer-term beneficial effects.^36,39,40^ At the young age of the children in this study, parental behaviors are still the largest determinant in shaping healthy behaviors.^41,42^ For example, evidence suggests that children’s engagement with greenness had a positive impact on their well-being and health later in life,^40^ which may be protective against multiple adverse health outcomes, including overweight and obesity.^36^

Our results also reflect the broader challenge of translating satellite-derived EVI measures into practical guidance for planners and policymakers. Current satellite data may not adequately capture local variations (<300 m) in public green spaces, where multiple features—such as parks, roadside trees, or allotments—may coexist. Clarifying the distinction between these measures may enable researchers, policymakers, and practitioners to better understand exposures and the mechanisms influencing health outcomes, particularly for young children.

Findings suggest that regardless of the measure of residential greenness, there are other factors that influence the association between greenness exposure and children’s likelihood of being overweight or obese. Our results emphasize that childhood weight status is influenced by many factors, such as environmental and socioeconomic characteristics, in complex ways. Green areas could be health‐promoting; however, EVI may not be a representative measure of greenness exposure in rural areas where deprivation levels might also be higher and therefore offset the health benefits derived from the natural greenness. We conclude that the association between residential greenness exposure and children’s weight status is complex and requires continued investigation.

## Conflicts of interest statement

The authors declare that they have no conflicts of interest with regard to the content of this report.

## ACKNOWLEDGMENTS

We would like to acknowledge all data providers who make anonymised data available for research. All research conducted has been completed under the permission and approval of the SAIL independent Information Governance Review Panel (IGRP) project number 1001.

## Supplementary Material
