# Supplementary material for: Cumulative residential greenness and childhood body mass index
Source: Environ Epidemiol. 2025 Sep 25;9(5):e421. doi: 10.1097/EE9.0000000000000421 (PMC12466902; doi:10.1097/EE9.0000000000000421)
Supplement: Supplementary file 1 [file ee9-9-e421-s001.pdf]

Supplementary Material

S-Table 1: Average number of green spaces within 800m of a child's residence, Wales, UK, 2012/13 – 2018/19

Average number of green spaces[a] within 800m of a child's residence	N	%
0	32,992	16.5
1	29,508	14.7
2	30,692	15.3
3	25,437	12.7
4	20,914	10.4
5	15,100	7.5
6	12,691	6.3
7	9,350	4.7
8	6,795	3.4
9	5,030	2.5
10	3,224	1.6
11	2,733	1.4
12	1,672	0.8
13	1,237	0.6
14	929	0.5
15	752	0.4
16	429	0.2
17	288	0.1
18	241	0.1
19	79	0
20	32	0
21	27	0
22+	85	0
Total	200,237	100

[a] green spaces with a pedestrian or vehicle access point within 800m of residence

S-Table 2: Differences in children's BMI weight category based on average EVI, Wales, UK, 2012/13 – 2018/19

	Healthy weight Odds Ratio (95% CI)		Overweight Odds Ratio (95% CI)		Obese Odds Ratio (95% CI)	
**Average EVI**	1.20	(1.15 – 1.25)	0.98	(0.95 – 1.01)	0.96	(0.85 – 1.09)
**Age (years)**						
4	-		-		-	
5	0.96	(0.95 – 0.96)	1.00	(0.99 – 1.01)	1.01	(0.99 – 1.03)
**Gender**						
Male	-		-		-	
Female	1.03	(1.02 – 1.03)	1.00	(0.99 – 1.01)	0.91	(0.89 – 0.93)
**Deprivation quintile (WIMD)**						
1 Most deprived	-		-		-	
2	1.01	(0.99 – 1.02)	1.00	(0.99 – 1.01)	0.97	(0.94 – 1.00)
3	0.99	(0.98 – 1.00)	0.99	(0.98 – 1.00)	0.97	(0.94 – 1.00)
4	0.98	(0.96 – 0.99)	0.99	(0.98 – 1.00)	0.92	(0.89 – 0.95)
5 Least deprived	0.93	(0.92 – 0.94)	0.98	(0.98 – 0.99)	0.88	(0.85 – 0.91)
**Urbanicity**						
Rural	-		-		-	
Urban	0.96	(0.95 – 0.97)	1.00	(0.99 – 1.01)	1.00	(0.97 – 1.02)

[a] Odds ratio per 1 unit increase in EVI (i.e. 0.2 to 0.3). CI = confidence interval

S-Table 3: Differences in children's BMI category based on average EVI by urban and rural categorisation, Wales, UK, 2012/13 – 2018/19

	Urban		Rural	
	**Odds Ratio[a] (95% CI)**		**Odds Ratio[a] (95% CI)**	
**Average EVI**	1.47	(1.25 – 1.74)	0.9	(0.73 – 1.11)
**Age (years)**				
4	-		-	
5	0.96	(0.93 – 1.00)	0.87	(0.82 – 0.93)
**Deprivation quintile (WIMD)**				
1 Most deprived	-		-	
2	0.93	(0.90 – 0.96)	0.89	(0.82 – 0.96)
3	0.88	(0.84 – 0.91)	0.79	(0.74 – 0.85)
4	0.78	(0.75 – 0.82)	0.75	(0.70 – 0.81)
5 Least deprived	0.62	(0.60 – 0.65)	0.58	(0.54 – 0.63)

[a] Odds ratio per 1 unit increase in EVI (i.e. 0.2 to 0.3). Outcome is overweight or obese; reference category is healthy weight. CI = confidence interval

S-Table 4: Differences in children's BMI category based on average EVI by gender, Wales, UK, 2012/13 – 2018/19

	Males		Females	
	Odds Ratio [a] (95% CI)		Odds Ratio [a] (95% CI)	
**Average EVI**	1.25	(1.05 - 1.50)	1.13	(0.94 - 1.35)
**Age (years)**				
4	-		-	
5	0.95	(0.92 - 0.98)	0.97	(0.94 - 1.00)
**Deprivation quintile (WIMD)**				
1 Most deprived	-		-	
2	0.95	(0.91 - 1.00)	0.90	(0.86 - 0.94)
3	0.86	(0.82 - 0.90)	0.84	(0.80 - 0.89)
4	0.78	(0.74 - 0.81)	0.78	(0.74 - 0.82)
5 Least deprived	0.62	(0.59 - 0.65)	0.62	(0.59 - 0.65)
**Urbanicity**				
Urban	-		-	
Rural	0.95	(0.91 - 0.99)	0.97	(0.93 - 1.01)

[a] Odds ratio per 1 unit increase in EVI (i.e. 0.2 to 0.3). Outcome is overweight or obese; reference category is healthy weight. CI = confidence interval
